# Structural and Functional Analysis of Phytotoxin Toxoflavin-Degrading Enzyme

**DOI:** 10.1371/journal.pone.0022443

**Published:** 2011-07-25

**Authors:** Woo-Suk Jung, Jeehyun Lee, Myung-Il Kim, Jun Ma, Tomohisa Nagamatsu, Eunhye Goo, Hongsup Kim, Ingyu Hwang, Jaehong Han, Sangkee Rhee

**Affiliations:** 1 Department of Agricultural Biotechnology, Seoul National University, Seoul, Korea; 2 Division of Pharmaceutical Sciences, Graduate School of Medicine, Dentistry and Pharmaceutical Sciences, Okayama University, Okayama, Japan; 3 Department of Biotechnology, Chung-Ang University, Anseong, Korea; 4 Center for Fungal Pathogenesis, Seoul National University, Seoul, Korea; University of Queensland, Australia

## Abstract

Pathogenic bacteria synthesize and secrete toxic low molecular weight compounds as virulence factors. These microbial toxins play essential roles in the pathogenicity of bacteria in various hosts, and are emerging as targets for antivirulence strategies. Toxoflavin, a phytotoxin produced by *Burkholderia glumae* BGR1, has been known to be the key factor in rice grain rot and wilt in many field crops. Recently, toxoflavin-degrading enzyme (TxDE) was identified from *Paenibacillus polymyxa JH2*, thereby providing a possible antivirulence strategy for toxoflavin-mediated plant diseases. Here, we report the crystal structure of TxDE in the substrate-free form and in complex with toxoflavin, along with the results of a functional analysis. The overall structure of TxDE is similar to those of the vicinal oxygen chelate superfamily of metalloenzymes, despite the lack of apparent sequence identity. The active site is located at the end of the hydrophobic channel, 9 Å in length, and contains a Mn(II) ion interacting with one histidine residue, two glutamate residues, and three water molecules in an octahedral coordination. In the complex, toxoflavin binds in the hydrophobic active site, specifically the Mn(II)-coordination shell by replacing a ligating water molecule. A functional analysis indicated that TxDE catalyzes the degradation of toxoflavin in a manner dependent on oxygen, Mn(II), and the reducing agent dithiothreitol. These results provide the structural features of TxDE and the early events in catalysis.

## Introduction

Pathogenic bacteria employ an array of protein molecules and/or secondary metabolites as mediators of pathogenicity in human, animal, and plant hosts. In general, while bacterial molecules such as lipopolysaccharides, capsular polysaccharides, peptidoglycans, and lipoproteins serve as pathogen-associated molecular patterns to initiate interactions with the host, they also activate the host innate immune system [1], [2]. To evade host defense systems, pathogens directly inject bacterial proteins, known as effectors, into the cytosolic compartment of host cells *via* various secretion systems [3], which have been well characterized in plant immune responses [1]. Effectors play essential roles in pathogenesis by disturbing the metabolism of host cells through several different strategies [3]–[5]. In addition to these recently characterized processes, bacteria have also been shown to synthesize and secrete toxic low molecular weight chemicals, collectively called toxins, as virulence factors [6]. The modes of action, which are unique to each toxin, show great diversity in host cells and range from gene regulation to the control of ion channel activity [6], [7]. Structural and single-molecule studies of toxins in complexes with target proteins have provided molecular insights into the functional roles of those toxins [8], [9].

**Figure 1:**
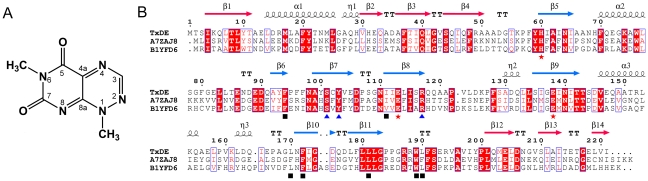
Chemical structure of toxoflavin and sequence alignment of TxDE. **A**, The chemical structure of toxoflavin is shown with the numbering of atoms. **B**, The amino acid sequence of TxDE and similar proteins are compared: TxDE from *Paenibacillus polymyxa* JH2 (GenBank accession number: GQ9218340) [18] and uncharacterized proteins from *Bacillus amyloliquefaciens* (A7ZAJ8) and *Exiguobacterium sibiricum* (B1YFD6). Highly conserved residues are shown in red and boxed in blue; strictly conserved residues are shown on a red background. Three residues that interact with Mn(II) in the active site are indicated by red asterisks; blue triangles represent the residues involved in hydrogen bonding at the active site; and black squares indicate residues having van der Waals interactions with toxoflavin. The secondary structural elements are shown for the corresponding sequences, with the A-domain in red and the B-domain in blue. This figure was prepared using ESPript [36].

In recent years, it has become evident that the expression of genes involved in the synthesis and secretion of toxins is regulated at the level of transcription by quorum sensing, the central mechanism for bacterial intercellular signaling that utilizes diffusible small chemicals as a signal [10]. This general theme is applicable to the phytopathogenic bacterium *Burkholderia glumae* BGR1, which is responsible for rice grain rot and wilt in many field crops. *B. glumae* produces toxoflavin (Figure 1A) [11], which acts as a key factor in this disease, possibly by producing superoxide and hydrogen peroxide [12], [13]. In fact, the synthesis and transport of toxoflavin in *B. glumae* were shown to be coordinately regulated by quorum sensing signals [14]. Therefore, interference of quorum sensing referred to as quorum quenching has been suggested as the antivirulence strategy, in which *N*-acylhomoserine lactones (AHLs), the quorum sensing signals produced in many Gram-negative bacteria, were degraded [15]. For example, lactonases produced by some *Bacillus spp.* are known to hydrolyze the lactone bond in the homoserine ring of AHLs, and transgenic plants expressing *Bacillus* AHL lactonase showed resistance to quorum sensing-dependent bacterial infection [16]. Recently, another type of quorum-quenching enzyme was identified, and structural analysis indicated that this enzyme hydrolyzes the peptide bond between the acyl chain and homoserine ring of AHLs [17].

In addition to quorum quenching, degradation of the toxin itself could serve as an antivirulence strategy in toxin-mediated diseases. An enzyme that showed the ability to degrade toxoflavin *in vitro* was recently identified from *Paenibacillus polymyxa JH2* and was named toxoflavin-degrading enzyme (TxDE) [18]. Although the *in vivo* function of TxDE in *P. polymyxa JH2* remains to be determined, its identification and characterization may contribute significantly to plant biotechnology by providing a non-antibiotic selection marker for plants [18]. Furthermore, this may lead to the development of disease-resistant crops carrying an antivirulence factor to prevent rice grain rot. To understand the structural and functional features of TxDE, we determined its crystal structure in substrate-free form and in complex with the substrate toxoflavin, and performed functional analysis of the active site residues. Further NMR study was also carried out in order to characterize the early event in catalysis.

## Results

### Overall Structure of TxDE

We have extensively searched for TxDE mutants suitable for structural study, because a wild-type enzyme failed to produce a crystal. Among those mutant enzymes, TxDE(F94S) successfully yielded a crystal for the initial structural analysis. The structure of TxDE was determined at 2.2 Å resolution using a crystal of selenomethionine (SeMet)-substituted TxDE(F94S) mutant enzyme (Table S1). However, the replacement of Phe-94 with a serine caused the enzyme to be catalytically inactive (Figure S1), possibly by hindering the binding of substrate. Subsequently, the TxDE(D175A) mutant, which is catalytically active (Figure S1), was crystallized. Its structure was determined in substrate-free form at 1.6 Å and in a binary complex with the substrate toxoflavin at 2.0 Å resolution, by molecular replacement with the refined structure of TxDE(F94S) as a search model (Table S1). These two structures have one monomer in an asymmetric unit. The structural features described in the present study are based on the structural analysis of TxDE(D175A).

**Figure 2:**
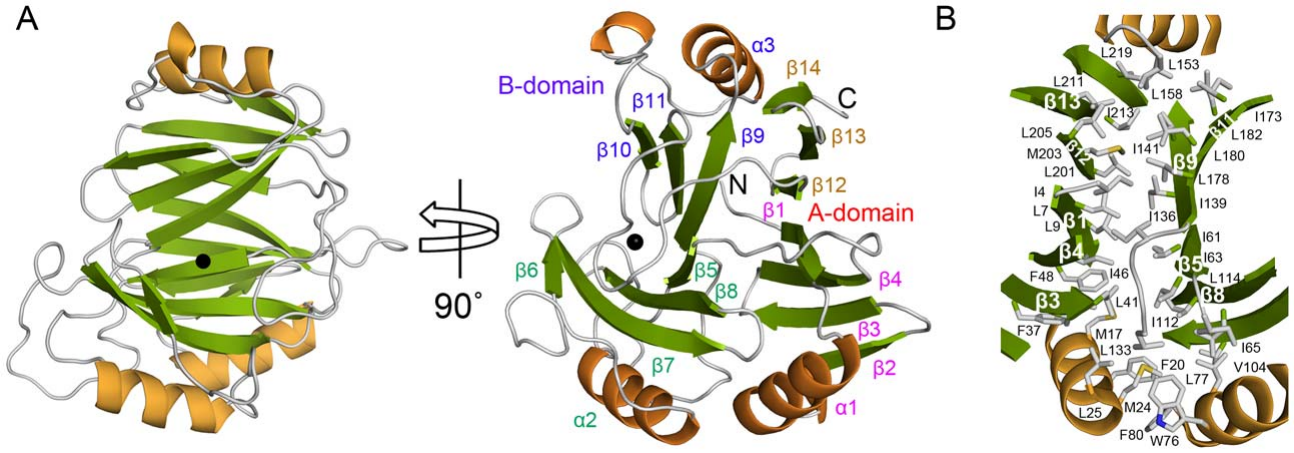
Overall structure of TxDE in the substrate-free form. **A**, A ribbon diagram is shown, with the corresponding secondary structures labeled as defined in Figure 1B. The black sphere indicates the Mn(II) ion. In the left panel, the molecule is oriented to place the B-domain in front, and a different view is shown in the right panel. Labels for the secondary structures in each motif are indicated in different colors. **B**, Hydrophobic interactions between the A- and B-domains are shown. Hydrophobic residues such as leucine, isoleucine, and phenylalanine are predominantly located in this interdomain interface. These residues along the β-sheet include Ile-213, Leu-211, Leu-205, Met-203, Leu-201, Ile-4, Leu-7, Leu-9, Ile-46, Phe-48, and Leu-41 from the A-domain, and Ile-173, Leu-178, Leu-180, Leu-182, Ile-136, Ile-139, Ile-141, Ile-61, Ile-63, and Ile-112 from the B-domain.

TxDE encodes 221 residues, but a total of 222 residues from Thr-2 to the extraneous leucine and glutamine at the C-terminus form 14 β-strands and three α-helices in the substrate-free form (Figure 1B). The topology of TxDE is composed mainly of two layers of β-sheets, which bisect the molecule into A- and B-domains, each with seven β-strands (Figure 2A). Specifically, the A-domain (residues 1–51 and 200–221) contains one βαβββ motif for the N-terminal 51 residues and one βββ motif for the C-terminal residues (Figure 1B). The strands in the A-domain are arranged in a mixed orientation, such that the β1 strand of the N-terminal β1^+^ β4^+^ β3^−^ β2^+^ motif (the superscripts + and − indicate parallel or antiparallel orientation relative to the β1 strand) makes edge-to-edge contacts in an antiparallel manner with the β12 strand of the C-terminal β12^−^ β13^+^ β14^−^ motif, resulting in a continuous seven-stranded β-sheet with a distorted orientation (Figure 2A). The only α-helix in the A-domain, α1, is located at the inner wall of the β-sheet, close to β2 and β3. A similar configuration, but with much greater distortion of the β strands, was observed in the B-domain (residues 58–186), which is connected to the A-domain by a long loop between β4 and β5, and between β11 and β12. Strands from β5 to β8 and helix α2 constitute a βαβββ motif, whereas three strands, β9 to β11, and helix α3 form a βαββ motif. Similar to the A-domain, two β strands in the B-domain, one from each motif (*i.e.*, β5 and β9), interact with each other in an edge-to-edge orientation, such that seven strands in two motifs are arranged in the spatial order β6^+^ β7^−^ β8^+^ β5^+^ β9^−^ β11^−^ β10^+^. The two motifs in the B-domain are almost orthogonal to each other, forming a highly concave surface along the seven-stranded β-sheet (Figure 2A). Helices α2 and α3 in the B-domain are positioned respectively at each end of the sheet.

### Interactions between the Two Domains

The overall structure of TxDE is stabilized by interactions between the two domains. In particular, the two β-sheets are packed in a parallel manner, such that the side chains of β-strands in the A-domain interact closely with those in the B-domain. These interactions are mediated mainly by hydrophobic residues. Specifically, leucine and isoleucine residues are predominantly located at the interface between the two domains: β3, β4, β1, β12, β13 from the A-domain and β11, β9, β5, β8 from the B-domain (Figure 2B). These interactions are so hydrophobic and extensive that water molecules are not present between the two layers of β sheets, with a total of 864 Å^2^ of the buried surface area, corresponding to 26% of the surface area of each sheet. In addition, the three helices, located at both ends of the β-sheet, seal off the potential cavity in the interdomain interface (Figure 2B). Specifically, residues in α1 (Met-17, Phe-20, Met-24, and Leu-25) and in α2 (Trp-76, Leu-77, and Phe-80) form hydrophobic interactions with residues in the A-domain (Phe-37, Leu-39, Leu-41, and Phe-48) and in the B-domain (Val-104 and Leu-114), along with a loop (Ile-65 and Leu-133) connecting the A- and B-domains. At the other end of the β-sheet, Leu-153 from α3 participates in interactions with Leu-219 of the A-domain, as well as Leu-158, Ile-173, and Leu-182 of the B-domain. All of these residues are within 4 Å of each other.

### Active Site in the Substrate-Free TxDE

**Figure 3:**
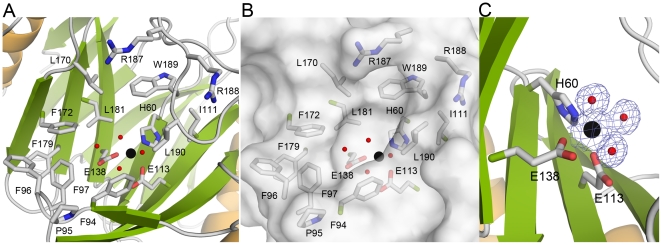
The active site of TxDE in the substrate-free form. **A**, A close-up view of the active site in the B-domain shows a deep, concave, funnel-like surface with nearby hydrophobic residues; the Mn(II)-binding site is indicated by the black sphere. **B**, Surface representation of (**A**) shows that the metal binding site is located deep within a hydrophobic channel. **C**, The active site metal has an octahedral coordination, with three amino acid ligands and three water molecules. The *2Fo-Fc* electron density map contoured at 1σ clearly indicates the locations of the metal and the three water molecules, represented by black and red spheres, respectively.

The location of the active site in TxDE was suggested by the presence of a possible metal binding site in the B-domain (Figure 2A), consistent with the requirement of a Mn(II) ion for its catalytic activity (Figure S1) [18]. We had also noted that TxDE became stable and soluble in the presence of Mn(II) ions; therefore, Mn(II) was supplemented at the early stage of protein purification. The metal binding site is embedded in the hydrophobic cavity in the B-domain, which was generated by a deep, concave, funnel-like surface enclosed by the hydrophobic residues. Unlike that in the A-domain, the funnel-like space in the B-domain has two possible openings, one at each end of the long axis of the funnel; however, these openings are effectively sealed off by mainly hydrophobic residues, including Phe-96, Phe-97, Phe-172, and Phe-179 at one end, and Ile-111, Arg-187, Arg-188, Trp-189, and Leu-190 at the other (Figure 3A). Although the top and bottom of the funnel are mostly closed off, there is a vent on the side of the funnel that connects the surface of the molecule to the inside of the enzyme (Figure 3B). This channel is elliptical, with dimensions of 8×10 Å on the basis of interatomic distance, and its wall is lined with hydrophobic residues: Phe-94, Pro-95, Phe-96, Phe-97, Ile-111, Leu-170, Phe-172, Leu-181, Trp-189, and Leu-190 (Figure 3B). The metal binding site is located at the end of the 9 Å-long hydrophobic channel from the vent.

In the metal binding site, three residues from the inner wall of the funnel (His-60 from β5, Glu-113 from β8, and Glu-138 from β9) as well as three well-ordered water molecules coordinate with the bound metal (Figure 3C). These six ligands form a coordination shell of octahedral geometry, with an average distance of 2.2 Å to the bound metal (Table S2). In this coordination shell, His-60 and Glu-138 form part of the equatorial plane with two water molecules, each across from His-60 and Glu-138, respectively, while Glu-113 and a water molecule *trans* to Glu-113 serve as the axial ligand. In addition to these metal-coordinating water molecules, there are several water molecules near the first coordination shell of the metal ion in the active site, within hydrogen bonding distance. The metal ion was identified as Mn(II) by the characteristic hyperfine signals from electron paramagnetic resonance spectroscopy (Figure S2). Therefore, it was concluded that TxDE is a metalloenzyme requiring Mn(II) for its activity, and this is consistent with the results of a functional analysis (Figure S1) [18].

### Structural Features of the TxDE–Toxoflavin Complex

The structure of TxDE(D175A) in complex with toxoflavin, designated as TxDE(D175A)–Tox complex, was determined in a soaking experiment. Structural superposition of substrate-free TxDE(D175A) and its complex with toxoflavin, performed using the CCP4MG molecular graphics program [19], did not indicate any noticeable differences in conformation between the two forms, which had a root mean square deviation of 0.33 Å for all Cα atoms.

**Figure 4:**
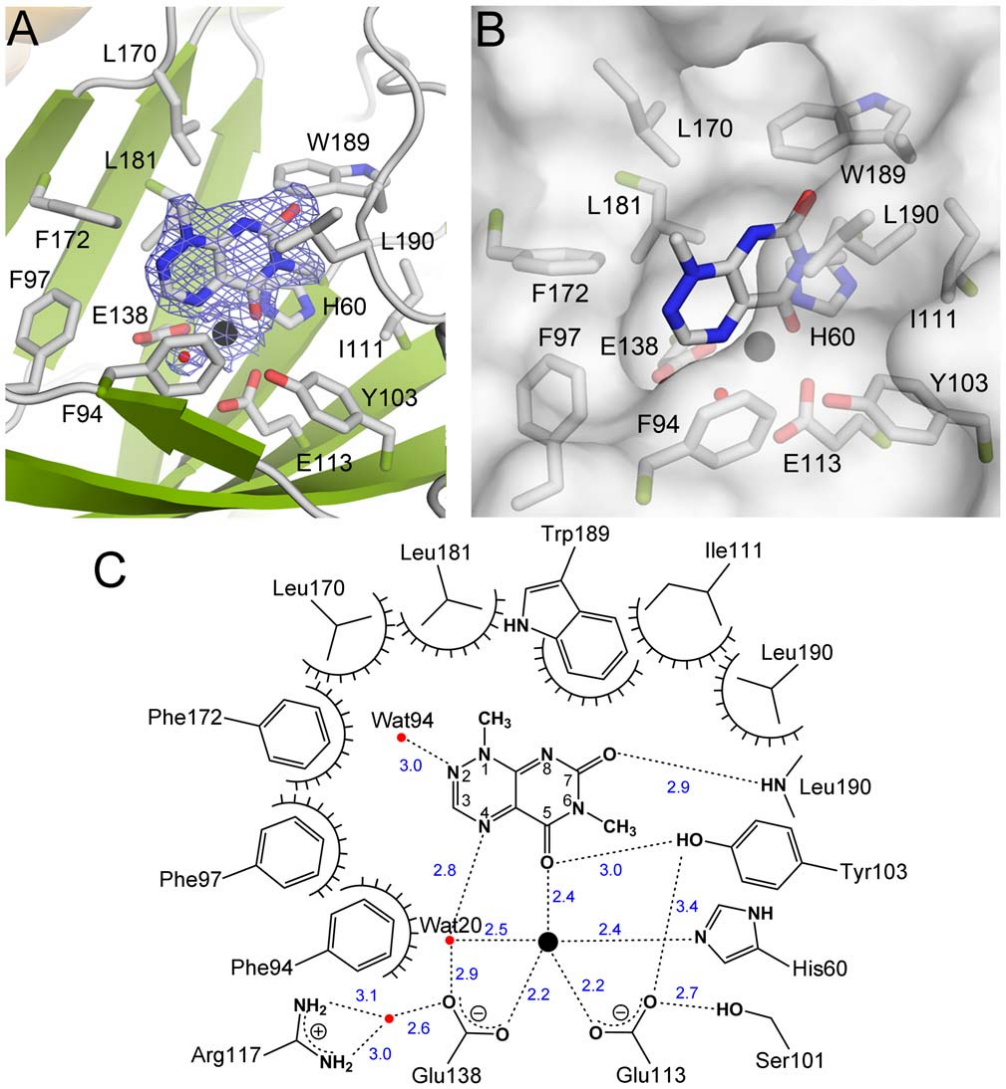
The active site in the TxDE–Tox complex. **A**, The binding of toxoflavin at the active site is shown, with the *2Fo-Fc* electron density map contoured at 1σ for a bound Mn(II) ion (black sphere), water molecule (red sphere), and toxoflavin molecule. **B**, The surface representation of the active site provides a better view of the toxoflavin molecule in the hydrophobic channel. **C**, Schematic diagram showing the binding mode of toxoflavin in the active site. The dashed lines indicate putative hydrogen bonds, which are labeled with the interatomic distance (in Å); the decorated arcs represent van der Waals interactions of less than 5.0 Å. Water molecules and a bound Mn(II) ion are shown as red and black spheres, respectively.

Figure 4 shows the details of the active site in the TxDE(D175A)–Tox complex. The substrate toxoflavin is bound to the Mn(II)-coordination shell, with an orientation such that the planar ring of toxoflavin fits into the long axis of the elliptical channel, and the methyl group at N1 points toward a vent (Figure 4, A and B). The binding site is surrounded by mainly hydrophobic residues in the wall of the channel: Phe-94, Phe-97, Ile-111, Leu-170, Phe-172, Leu-181, Trp-189, and Leu-190. Specifically, O5 of toxoflavin replaced the Mn(II)-ligating water molecule across from Glu-138 of the equatorial plane in the substrate-free structure, at a distance of 2.4 Å relative to the Mn(II) ion, whereas another equatorial water molecule *trans* to His-60 remained in the coordination shell (Figure 4A and Figure S3). However, the axial water molecule, which was present in the substrate-free form, was absent in the active site of the complex. The planar ring of toxoflavin was at an angle of about 55° relative to the equatorial plane of the coordination shell; thus the axial ligand, if present, would have been only about 2.8 Å from the toxoflavin ring (Figure S3). Other water molecules present in the substrate-free form were also replaced; in particular, the methyl groups at N6 and O7 now occupy the positions of water molecules in the substrate-free form.

The binding of toxoflavin in the complex appears to be stabilized by hydrophobic interactions and hydrogen bonds (Figure 4, A and C). Specifically, the side chains of Phe-94, Phe-172, Trp-189, and Leu-190 are within 4.0 Å of toxoflavin, with the side chains of Phe-94 and Leu-190 positioned parallel to the toxoflavin ring at a distance of 3.6–3.8 Å (Figure 4A and Figure S3). Other hydrophobic residues such as Phe-97, Leu-170, and Leu-181 are involved in the formation of a hydrophobic environment over a range of 4–5 Å from toxoflavin. In addition to these hydrophobic interactions, there are several hydrogen bonds between toxoflavin and its neighboring residues: O5 to the hydroxyl group of Tyr-103 at 3.0 Å, O7 to the main chain nitrogen of Leu-190 at 2.9 Å, and N2 to a water molecule at 3.0 Å. It is noteworthy that the side chains of Trp-189 and Leu-190 are oriented such that part of the toxoflavin is sandwiched into the cavity between these two side chains (Figure 4A and Figure S3). This binding mode is likely to be stabilized by hydrophobic interactions and a hydrogen bond between O7 and the main-chain nitrogen of Leu-190, as described above.

### Functional Analysis

To understand the functional roles of residues in the active site, we performed thin-layer chromatographic analysis to measure the degradation of toxoflavin after reaction with various mutant enzymes. In fact, our attempts to measure the kinetic parameters of the wild-type and each mutant enzyme were unsuccessful using UV-Visible spectroscopic and thin-layer chromatographic analysis, mainly due to the complexity of the reaction (see *Discussion*), as well as the detection limits of thin-layer chromatographic analysis.

**Figure 5:**
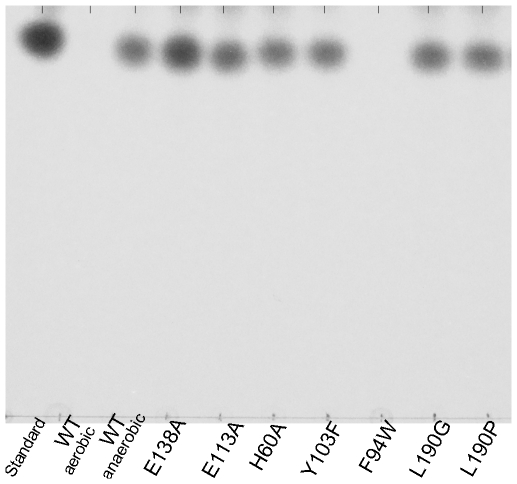
Thin-layer chromatographic analysis of toxoflavin degradation under various conditions using wild-type and mutant TxDEs. Reactions were performed at 25°C under aerobic conditions, unless specified. Chemically synthesized toxoflavin is shown as the Standard. The wild-type enzyme was highly inactive under anaerobic conditions. Enzymes with mutations of the metal-binding residues E138A, E113A, or H60A or of other residues near the active site (Y103F, L190G, or L190P) were catalytically incompetent relative to the wild-type enzyme. Note that some variations of intensity for the residual toxoflavin after a reaction are mainly due to the recovery yield, while chemically synthesized toxoflavin was used as a standard without a reaction.

First, we examined whether TxDE catalyzes toxoflavin degradation in an oxygen-dependent manner, because our analysis indicated that TxDE is structurally similar to oxygen-dependent enzymes (see *Discussion*). The assay results indicated that substantial amounts of toxoflavin remained under anaerobic conditions, in contrast to aerobic conditions (Figure 5), strongly supporting the suggestion that TxDE requires oxygen, as well as Mn^2+^ and the reducing agent DTT, for the degradation of toxoflavin (Figure S1) [18]. Second, the residues in the Mn(II)-binding site (Figure 3C) were also shown to be essential for the catalytic activity of the enzyme (Figure 5), consistent with the metal requirement for enzyme activity. In addition, we showed that the hydroxyl group of Tyr-103 and the hydrophobic features of Leu-190 and Phe-94 play a crucial role in enzyme catalysis.

## Discussion

Low molecular weight toxins secreted from pathogenic bacteria exert destructive effects as virulence factors in various hosts; the modes of action for fungal toxins have been reviewed [7]. The identification of enzymes capable of degrading these toxins may provide an alternative antivirulence strategy for toxin-mediated diseases. To facilitate the investigation of the catalytic features of toxoflavin-degrading enzyme, we report the crystal structure of TxDE from *P. polymyxa* JH2. This enzyme exhibits *in vitro* degradation activity toward phytotoxin toxoflavin, a virulence factor produced by the pathogenic bacterium *B. glumae* [14], [18]. Structural analysis of the substrate-free form of TxDE and its complex with the substrate toxoflavin, as well as the results of a functional analysis, suggest the unique features of the enzyme.

TxDE appears to be novel in its *in vitro* function, as no other enzymes with a similar function have been characterized to date; however, hypothetical proteins with sequence similarities of 52–57% with TxDE were identified from various *Bacillus* species (37% identity and 57% similarity) and *Exiguobacterium sibiricum* (38% identity and 52% similarity), which were annotated as members of the glyoxalase family (Figure 1B) (see below). A structural alignment search using DALI [20] indicated that TxDE is structurally similar to a functionally uncharacterized protein from *B. cereus* (Z-score, 12.2; sequence identity, 11%; PDB ID 1ZSW from Midwest Center for Structural Genomics) and has limited structural similarity with other functionally known proteins, despite a low degree of sequence similarity (6–25%) and low Z-score (4.8–8.6). In fact, all of these structurally similar proteins are members of the vicinal oxygen chelate superfamily of metalloenzymes [21], [22], which includes glyoxalase, bleomycin resistance protein, fosfomycin resistance protein, methylmalonyl-coenzyme A epimerase, and bacterial extradiol dioxygenases. Proteins in this family share a common structural feature in which the βαβββ motif is the basic structural module of their three-dimensional conformation, although the numbers and relative orientations of these modules in the monomer and their oligomerization vary among the proteins [21], [22]. Specifically, glyoxalase and bleomycin resistance protein in this family contain two βαβββ motifs in each monomer and form a functional dimer; the edge-to-edge interactions of β-strands between motifs in different monomers generate two independent active sites at the intersubunit interface (Figure S4A). In contrast, 2,3-dihyroxybiphenyl 1,2-dioxygenase (DHBD) from *Pseudomonas cepacia* [23] is similar to TxDE in that there are four motifs in the monomer, with sequentially ordered motifs mediating edge-to-edge interactions and forming two domains, and the active site is present in only one particular domain (Figure S4B). From a structural perspective, the topology of TxDE differs from that of the three proteins described above; four motifs in the monomer that form a continuous β-stand in two domains are not assembled in a sequential manner, but rather with edge-to-edge interactions between motifs 1 and 4, and motifs 2 and 3 (Figure 2).

The structural similarity with DHBD is extended to the active site of TxDE, in which one histidine and two glutamate residues along with three water molecules serve as the first coordination shell for Mn(II) (Figure 3C and Table S2). In the extradiol dioxygenases, to which DHBD belongs, two histidines and one carboxylate with three water molecules is a common motif of the active site bearing Fe(II) or Mn(II), to catalyze ring cleavage of catecholic compounds by activating and incorporating dioxygen into the substrate, producing a ring-opened product [24], [25]. Consistent with this structural similarity, mutation of the Mn(II)-coordinating residues His-60, Glu-113, and Glu-138 (Figure 5), as well as the absence of Mn(II) from the reaction medium, abolished TxDE enzyme activity (Figure S1). It was also noticed that in contrast to aerobic conditions, the substrate toxoflavin was not efficiently degraded under anaerobic conditions (Figure 5). Taken together, these observations suggest that TxDE catalyzes the degradation of toxoflavin in an oxygen- and Mn(II)-dependent manner.

Our functional analysis also indicates that the identity and precise orientation of active site residues play an essential role in catalysis, possibly by positioning the incoming toxoflavin into the productive binding mode for catalysis. Consistent with the structural implication of Phe-94 (Figure 4A), the F94W mutant enzyme, which could enhance stacking interactions with the substrate, maintained activity, whereas the F94S mutant was catalytically inactive (Figure 5 and Figure S1). Also, the absence of a hydrophobic feature in the L190G mutant and a possible distortion of the main chain in the L190P mutant almost abolished enzyme activity (Figure 5), supporting the proposed structural role of Leu-190 as a part of the substrate binding pocket (Figure 4). The functional role of the hydroxyl group in Tyr-103 remains to be established.

Another notable feature of TxDE is the requirement for the reducing agent DTT for catalysis (Figure S1) [18]. The results of UV-visible spectroscopic analysis suggested that toxoflavin is subject to chemical modification by DTT, even in the absence of the enzyme, to form reduced toxoflavin (peak at 244 nm) and oxidized DTT (*i.e.*, 1,2-dithiane-4,5-diol; peak at 287 nm) (Figure S5). Subsequent NMR experiments also validated that toxoflavin is converted to 4,8-dihydrotoxoflavin in the presence of DTT, with the concurrent formation of oxidized DTT (Figure S6). Further analysis indicated that the reduced form of toxoflavin indeed serves as a substrate for the enzyme (Figure S5). Therefore, DTT is a prerequisite for the formation of the reduced form of toxoflavin.

An unusual feature of TxDE-dependent catalysis is the reaction product(s). In a dioxygenase-catalyzed reaction, the product is a chemically stable muconic semialdehyde adduct [24], [25]. However, there is no solid evidence for the stable products from TxDE-dependent degradation of toxoflavin; instead, many molecules with a diverse range of molecular mass, but lower than that of toxoflavin, were characterized by LC-MS analysis. This strongly suggests that TxDE produces a chemically labile molecule which cannot be characterized under our LC-MS analysis, and the unstable molecule(s) is likely subject to successive reactions in a spontaneous or/and enzyme-dependent manner. In fact, the reduced form of toxoflavin, not the oxidized form, was shown to be subject to oxidation and decarboxylation [26], [27]. Therefore, we postulate that toxoflavin is reduced by DTT and subsequently 4,8-dihydrotoxoflavin is subject to oxidation for further reaction which is not yet characterized. Owing to the complexity of the TxDE reaction, the detailed features with regard to the catalytic mechanism and final product by TxDE remain to be elucidated. Further investigations are required to answer details of the degradation pathway.

In this study, we determined the crystal structure of TxDE, an enzyme that exhibits *in vitro* degradation activity against the phytotoxin toxoflavin. Structural and functional analyses indicate that the enzyme is similar to a dioxygenase in both its structure and function, and that toxoflavin degradation is catalyzed in an oxygen-, DTT-, and Mn(II)-dependent manner. The characterization of TxDE may facilitate the development of disease-resistant crop plants as well as applications in other areas of biotechnology [18].

We note the recent publication of Fenwick *et al.* [28] describing the crystal structure of TxDE. Structural and functional features of TxDE reported in these two independent studies are almost identical.

## Materials and Methods

### Construction of TxDE Variants

For structural and functional analysis, various TxDE enzymes were produced as described below. The DNA fragment of TxDE was amplified from cDNA of *Paenibacillus polymyxa* JH2 [18] (GenBank accession number GQ921834) by PCR with sequence-specific and/or mutagenic primers. The resulting PCR products were ligated into the *Nde*I and *Xho*I sites of the expression vector pET41b containing a C-terminal His-tag. Since a wild-type TxDE failed to produce a crystal for structural analysis, extensive search has been carried out to identify TxDE mutants suitable for further structural study. Among those mutant enzymes produced, TxDE(F94S) successfully yielded a crystal for the initial structural analysis. Subsequently, the TxDE(D175A) mutant was used for further structural analysis of the substrate-free form and the complex with the substrate toxoflavin.

### Expression, Purification, and Crystallization

*Escherichia coli* BL21(DE3) *pLys*S strain (Stratagene) harboring the plasmid was used to express the C-terminal His-tagged TxDE protein. Cells were grown at 37°C in Luria-Bertani medium containing 10 mg/L kanamycin and 34 mg/L chloramphenicol to an OD_600_ of 0.8, and then induced at 37°C for 4 h with the addition of 1 mM isopropyl-1-thio-β-d-galactopyranoside. The harvested cells were sonicated in buffer A (50 mM Tris-HCl, pH 7.5, 200 mM NaCl, 1 mM DTT, and 1 mM MnCl_2_) and subjected to centrifugation at 30,000×*g* for 1 h. The crude extract was applied to a HisTrap column (GE Healthcare), and TxDE protein was eluted using buffer B (buffer A plus 500 mM imidazole). After dialysis against buffer C (50 mM Tris-HCl, pH 7.5, 1 mM DTT, and 1 mM MnCl_2_), the protein was further purified using a Mono-Q column (GE Healthcare) with a linear gradient of NaCl. After dialysis against buffer C, the enzyme was concentrated to about 12 mg/mL for crystallization. A SeMet-substituted TxDE(F94S) protein was prepared as described above, except that the expression plasmid was transformed into *E. coli* strain B834(DE3)*pLys*S, a methionine auxotroph (Novagen), and the protein was expressed in minimal medium in the presence of 10 µg/mL SeMet.

Crystallization was carried out at 22°C using the sitting-drop vapor-diffusion method. Crystals of TxDE(F94S) were produced with buffer containing of 0.1 M MES, pH 6.5, 50 mM CaCl_2_, 28% (v/v) PEGMME5000, and 0.1 M NaI, whereas TxDE(D175A) crystals were produced under the following conditions: 0.1 M CAPS, pH 10.5, 0.2 M LiSO_4_, and 1.2 M NaH_2_PO_4_/0.8 M K_2_HPO_4_.

### Data Collection and Structure Determination

In general, crystals of SeMet-TxDE(F94S) and TxDE(D175A) were soaked in the respective crystallization mother solution with the addition of the appropriate cryoprotectant (see below) as well as ligand, as necessary, and then flash-frozen in liquid nitrogen. For structure determination by multi-wavelength anomalous dispersion, data using a SeMet-TxDE(F94S) crystal were collected at three different wavelengths to 2.2 Å resolution at 100 K. Later, single-wavelength data were also obtained using crystals of TxDE(D175A) to 1.6 Å resolution and the complex with toxoflavin at 2.0 Å resolution, respectively, on beamlines 4A and 6C at Pohang Accelerator Laboratory, Pohang, Korea. All crystals had the symmetry of the space group *R*3; however, owing to different cell parameters, there were four monomers and one monomer in an asymmetric unit for TxDE(F94S) and TxDE(D175A) crystals, respectively. Collected data were processed using the HKL2000 package [29] (Table S1).

Glycerol was initially used as a cryoprotectant in structural analysis of SeMet-TxDE(F94S) crystals. However, preliminary data indicated preferential binding of glycerol to the active site; therefore, either sucrose or PEG4000 was used as an alternative cryoprotectant in the structural analysis of TxDE(D175A) crystals. Specifically, the TxDE(D175A) crystal was soaked in a solution of 0.1 M CAPS, pH 10.5, and 48% PEG4000 as cryoprotectant for the substrate-free structure. As toxoflavin becomes very labile at high pH (particularly above pH 9.5), an extensive search was carried out for the formation of the complex with toxoflavin. Later, we found that the TxDE(D175A)-toxoflavin complex could be formed by soaking a TxDE(D175A) crystal for about 30 min in a solution of 0.1 M HEPES, pH 7.5, 2 M ammonium sulfate, 2% PEG400, and 20% sucrose, as well as additional 1 mM MnCl_2_, 5 mM DTT, and 2 mM toxoflavin.

For TxDE(F94S) structure determination, the program SOLVE/RESOLVE [30], [31] was used for initial phasing and density modification. The initial electron density map was sufficiently interpretable to trace all residues, except the N-terminal Met-1 and the last two histidine residues in the C-terminal His-tag. The model was built using O [32] and refined using CNS [33]. The *R*_work_ and *R*_free_ of the TxDE(F94S) structure were 25% and 31%, respectively, after refinement using CNS. Later, data for TxDE(D175A) at 1.6 Å resolution were collected, and the structure was determined by molecular replacement using the program CNS, with a refined model of monomeric structure from TxDE(F94S) as a search model. Model building and refinement were carried out in a manner identical to that for TxDE(F94S). The structure of the TxDE(D175A)–toxoflavin complex was also determined by molecular replacement using the program CNS, with a refined model of TxDE(F94S) as a search model. Currently, a structure of TxDE(D175A) and its complex with toxoflavin is refined to final *R*_work_ */R*_free_ values of 19.5/22.0% and 21.9/25.7%, respectively, and no residues were found to be in a disallowed region in Ramachadran plot, except for Gln-176. Details of the refinement are listed in Table S1.

The atomic coordinates and structure factors (codes 3OUL for the substrate-free form of TxDE(D175A), 3OUM for its complex with toxoflavin) have been deposited in the Protein Data Bank (http://www.rcsb.org).

### Functional Analysis

An enzyme assay to measure the degradation of toxoflavin was performed according to previously reported procedures [18]. All enzymes used were expressed and purified as described above, and their reactions were performed at 25°C under aerobic conditions, unless otherwise specified. Briefly, 400 µL of assay buffer (50 mM sodium phosphate, pH 7.0) contained 20 µL of TxDE enzyme (3 mg/mL), 50 µM toxoflavin, 10 µM MnCl_2_, and 5 mM DTT. After a 30-min incubation, an equal volume of chloroform was added to the assay buffer to stop the reaction. The resulting chloroform fraction was dried completely and then solubilized in 10 µL of methanol. Thin-layer chromatography was performed, and the degradation of toxoflavin was visualized under UV light at 365 nm.

For the enzyme reaction under anaerobic conditions, all processes were carried out in an anaerobic chamber filled with N_2_ (MO Tek, Korea). Specifically, the reagent solutions were prepared in the chamber, and the protein solution was degassed before transport to the chamber. For the reactions in the absence of DTT or Mn^2+^ (Figure S1), the purified enzyme was first extensively dialyzed against a buffer of 50 mM Tris, pH 7.5, and 10 mM EDTA, and then dialyzed again against 50 mM Tris buffer (pH 7.5).

### ^1^H-NMR Study

Pure toxoflavin [34], 4,8-dihydrotoxoflavin [11], DTT, and 1,2-dithiane-4,5-diol (DTD) [35] at a concentration of 10% in 99% deuterated methanol (CD_3_OD) were measured as the authentic compounds. The spectra (**A**) to (**C**) of Figure S6 show the peak assignments for each proton in toxoflavin, 4,8-dihydrotoxoflavin, and DTT, respectively. The reaction was carried out in NMR tubes with an internal diameter of 5 mm under aerobic conditions at 22°C, and all spectra were measured in 99% CD_3_OD. A mixture of toxoflavin (5 mg, 0.026 mmol) and (±)-DTT (4 mg, 0.026 mmol) in 99% CD_3_OD (5 mL) was left to stand for 10 min. Then, the spectrum of the mixture was measured at 22°C (Figure S6D). After oxygen was bubbled into the reaction mixture for 1 min, the spectrum of the mixture was obtained (Figure S6E).

The following are the ^1^H-NMR (in CD_3_OD) data for toxoflavin: δ 3.41 (3H, s, 6-Me), 4.09 (3H, s, 1-Me), 8.91 (1H, s, 3-H); for 4,8-dihydrotoxoflavin: δ 3.20 (3H, s, 6-Me), 3.45 (3H, s, 1-Me), 7.13 (1H, s, 3-H); for DTT: δ 2.63 (4H, d, *J*_1,2_ = *J*_3,4_ = 6.3 Hz, 1- and 4-CH_2_), 3.67 (2H, t, *J* = 6.0 Hz, 2- and 3-CH); for 1,2-dithiane-4,5-diol: δ 2.82–2.92 (2H, m, 3-H_a_ and 6-H_a_), 2.98–3.08 (2H, m, 3-H_b_ and 6-H_b_), 3.46–3.54 (2H, m, 4- and 5-H).

## Supporting Information

Table S1 **Crystallographic data and refinement statistics.**
(DOC)

Table S2 **Details for distances and angles (degrees) between a bound metal and its ligands.**
(DOC)

Figure S1 **Thin-layer chromatographic analysis of toxoflavin degradation under various conditions.** The enzyme reaction was carried out using three different enzymes: wild-type enzyme (WT), TxDE with the F94S mutation, and TxDE with the mutation D175A. For the reaction in the absence of DTT or Mn^2+^, the purified WT enzyme was dialyzed against buffer in the presence of 10 mM EDTA, and then DTT or Mn^2+^ was added. The “Standard” lane is toxoflavin in the absence of any other components. Toxoflavin was degraded by D175A mutant enzymes, but not by the F94S mutant enzyme, as well as in the absence of DTT or Mn^2+^. All reactions were carried out under aerobic conditions.
(TIF)

Figure S2 **EPR spectrum of the purified TxDE.** Sample contains 290 uM TxDE. EPR parameters: 100 K, 1-mW microwave power at 9.18 GHz, modulation amplitude 3.2G.
(TIF)

Figure S3 **Stereoscopic view of the active site of the TxDE–toxoflavin complex.** This view, obtained by a rotation of about 90° along the vertical axis of Figure 4**A**, illustrates that the possible sixth coordinating ligand is missing in this complex. The electron density of 2*Fo-Fc* contoured at 1 σ is shown for a bound Mn(II) (black sphere), water molecule (red sphere), and toxoflavin molecule.
(TIF)

Figure S4 **Overall structure of glyoxalase and 2,3-dihyroxybiphenyl 1,2-dioxygenase (DHBD).** (A) As described in the text, a dimer of glyoxalase (PDB ID 1FRO) [37] generates two independent active sites at the intersubunit interface. Each monomer is indicated in a different color, and the active sites are presented with a bound metal ion (black sphere). (B) The structure of monomeric DHBD (PDB ID 1HAN) [23] was similar to that of TxDE in this study. Each domain is colored differently. In each domain, two sequentially ordered βαβββ motifs form continuous β-stands by edge-to-edge interactions. The C-terminal active site is shown with a bound metal ion (black sphere).
(TIF)

Figure S5 **UV-Vis absorption spectra of toxoflavin in the absence and presence of DTT.** Two different absorption spectra of toxoflavin (25 µM), which was dissolved in 50 mM HEPES, pH 6.8, and 10 µM MnCl_2_, were recorded under aerobic conditions. In the absence of DTT (solid line), toxoflavin exhibits two absorption peaks, at 258 and 393 nm. Upon the addition of 2 mM DTT (dashed line), two peaks appeared, at 244 and 287 nm. The absorption peak at 287 nm corresponds to that of the oxidized form of DTT (*i.e.*, 1,2-dithiane-4,5-diol; DTD), and its absorbance varies according to the concentration of DTT used in the experiment. The peak at 244 nm was later identified by NMR spectroscopy as that of reduced toxoflavin (*i.e.*, 4,8-dihydrotoxoflavin) (Figure S6); it remained stable only in the presence of DTT. After the DTT was exhausted, the spectrum of 4,8-dihydrotoxoflavin changed into that of toxoflavin (solid line) owing to oxidation by adventitious air or bubbled oxygen, with an additional absorbance shoulder at 287 nm for DTD. At this stage, toxoflavin was no longer degraded by the TflA enzyme, unless additional DTT was added to the reaction mixture, strongly suggesting that the reduced form of toxoflavin is the true substrate for the enzyme.
(TIF)

Figure S6 **^1^H-NMR experiments in deuterated methanol (99% CD_3_OD).** ^1^H-NMR chemical shifts under aerobic conditions at 22°C for (A) pure toxoflavin (**1**), (B) pure 4,8-dihydrotoxoflavin (**2**), and (C) pure DTT (**3**) are shown with peak assignments for each proton in the compounds. (D) The ^1^H-NMR experiment was performed in deuterated methanol after a 10-min reaction of toxoflavin (**1**) with an equimolar amount of DTT (**3**) at 22°C under aerobic conditions. A chemical shift analysis indicated that the reaction mixture contained predominantly 4,8-dihydrotoxoflavin (**2**) and DTD (**4**) with a small quantity of DTT (**3**), consistent with the results of the UV-Vis spectroscopic analysis shown in Figure S5. (E) After the reaction of toxoflavin (**1**) with an equimolar amount of DTT (**3**) in deuterated methanol at 22°C for 10 min under aerobic conditions, oxygen was bubbled into the reaction mixture for 1 min. The analysis indicated that all DTT (**3**) was converted into DTD (**4**) and 4,8-dihydrotoxoflavin (**2**) was converted into toxoflavin (**1**) by oxidation, again consistent with the results of the UV-Vis spectroscopic analysis shown in Figure S5.
(TIF)
