# Supplementary material for: Structural and Functional Analysis of Phytotoxin Toxoflavin-Degrading Enzyme
Source: PLoS One. 2011 Jul 25;6(7):e22443. doi: 10.1371/journal.pone.0022443 (PMC3143149; doi:10.1371/journal.pone.0022443)
Supplement: Table S1 — Crystallographic data and refinement statistics. (DOC) [file pone.0022443.s001.doc]

**Table S1.** Crystallographic data and refinement statistics

|  | SeMet TxDE(F94S) | | | TxDE(D175A) | TxDE(D175A)-Tox |
| --- | --- | --- | --- | --- | --- |
| **Data collection** | Peak | Edge | Remote |  |  |
| Unit cell  a, b, c (Å)  α, β, γ (°) | a = b = 214.7  c = 57.1  α = β = 90, γ = 120 | | | a = b = 110.4  c = 56.8  α = β = 90, γ = 120 | a = b = 111.0  c = 57.4  α = β = 90, γ = 120 |
| Wavelength (Å) | 0.97948 | 0.97963 | 0.97179 | 1.2398 | 1.2398 |
| Resolution (Å) | 50.0-2.2 (2.28-2.2) | | | 50.0-1.6 (1.66-1.6) | 50.0-2.0 (2.03-2.0) |
| R_merge_^a^ (%) | 7.6 (42.6) | 6.1 (45.4) | 7.0 (58.2) | 6.4 (25.7) | 7.2 (30.1) |
| I/σI | 34.2 (5.1) | 34.9 (3.8) | 27.9 (2.0) | 30.9 (4.1) | 28.0 (5.0) |
| Completeness (%) | 96.9 (68.7) | 96.9 (69.2) | 98.8 (87.7) | 98.5 (88.5) | 100.0 (99.9) |
| Redundancy | 11.1 (10.3) | 7.7 (6.9) | 7.5 (5.4) | 6.1 (5.3) | 6.3 (5.9) |
| **Refinement** |  | | |  |  |
| Resolution (Å) | 50.0-2.2 | | | 50.0-1.6 | 50.0-2.0 |
| No. reflections (test) | 43,558 (4,371) | | | 30,441 (3,030) | 15,929 (1,552) |
| R_work_^b^/R_free_^c^ (%) | 24.9 / 31.0 | | | 19.5 / 22.0 | 21.9 / 25.7 |
| No. atoms |  | | |  |  |
| Protein | 7,024 | | | 1,738 | 1,738 |
| Ligand |  | | |  | 14 |
| Water | 159 | | | 134 | 102 |
| Avg. B-factor (Å^2^) |  | | |  |  |
| Protein | 36.3 | | | 22.7 | 25.8 |
| Ligand |  | | |  | 32.5 |
| Water | 31.7 | | | 28.5 | 32.3 |
| RMS deviations |  | | |  |  |
| Bond lengths (Å) | 0.007 | | | 0.010 | 0.009 |
| Bond angle (º) | 1.356 | | | 1,553 | 1.442 |
| Ramachandran plot |  | | |  |  |
| Most favored (%) | 81.6 | | | 88.3 | 89.8 |
| Allowed (%) | 18.2 | | | 11.2 | 9.7 |
| Disallowed | 0.2 | | | 0.5^d^ | 0.5 ^d^ |

Values in parenthesis are for the highest resolution shell.

^a^*R*_merge_ = Σ|*I*_h_ - 〈*I*_h_〉 |/Σ *I*_h_, where *I*_h_ is the observed intensity and 〈*I*_h_〉 is the average intensity.

^b^*R*_work_ = Σ||*F*_obs_| - k|*F*_cal_||/Σ|*F*_obs_|.

^c^*R*_free_ is the same as *R*_obs_ for a selected subset (10%) of the reflections that was excluded from refinement.

^d^Gln-176 is in a disallowed region.
