# Supplementary material for: Structural and Functional Analysis of Phytotoxin Toxoflavin-Degrading Enzyme
Source: PLoS One. 2011 Jul 25;6(7):e22443. doi: 10.1371/journal.pone.0022443 (PMC3143149; doi:10.1371/journal.pone.0022443)
Supplement: Table S2 — Details for distances and angles (degrees) between a bound metal and its ligands. (DOC) [file pone.0022443.s002.doc]

**Table S2.** Details for distances and angles (degrees) between a bound metal and its ligands.

(A) TxDE(D175A) in a substrate-free form


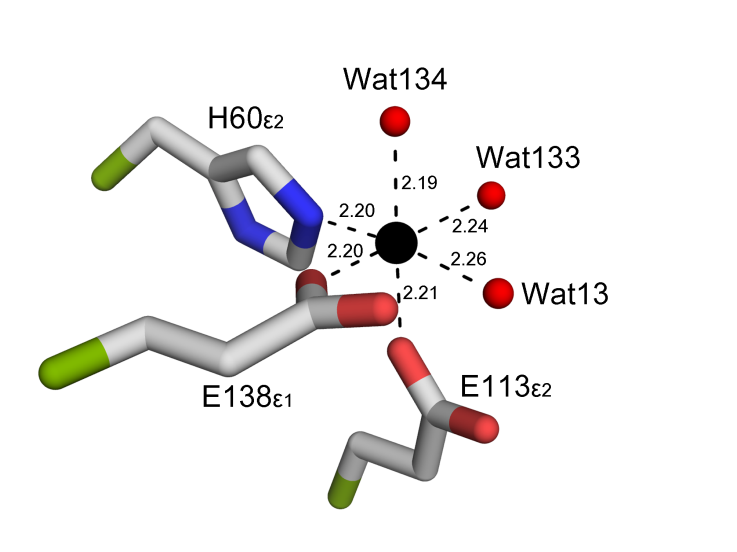


| Defining Atoms | Observed Angles  (degrees) | Defining Atoms | Observed Angles  (degrees) |
| --- | --- | --- | --- |
| H60N*^ε^*^2^-Mn-E138O*^ε^*^1^ | 96.6 | E113O*^ε^*^2^-Mn-Wat133 | 91.6 |
| H60N*^ε^*^2^-Mn- Wat13 | 170.4 | E138O*^ε^*^1^-Mn-E113O*^ε^*^1^ | 96.8 |
| H60N*^ε^*^2^-Mn-Wat133 | 94.9 | E138O*^ε^*^1^-Mn-Wat13 | 85.2 |
| E113O*^ε^*^1^-Mn-Wat13 | 87.0 | E138O*^ε^*^1^-Mn-Wat133 | 166.5 |
| E113O*^ε^*^1^-Mn-H60N*^ε^*^2^ | 83.4 | Wat133-Mn-Wat13 | 84.7 |
| E113O*^ε^*^1^-Mn-Wat134 | 175.0 | H60N*^ε^*^2^-Mn- Wat134 | 94.3 |
| E138O*^ε^*^1^-Mn-Wat134 | 87.9 | Wat133-Mn-Wat134 | 84.0 |
| Wat13-Mn-Wat134 | 95.2 |  |  |

(B) TxDE(D175A)-toxoflavin complex


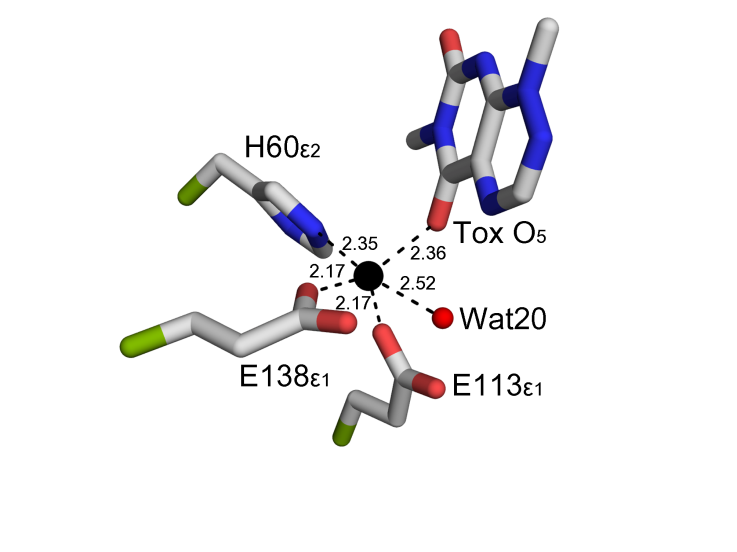


| Defining Atoms | Observed Angles  (degrees) | Defining Atoms | Observed Angles  (degrees) |
| --- | --- | --- | --- |
| H60N*^ε^*^2^-Mn-E138O*^ε^*^1^ | 100.9 | E113O*^ε^*^2^-Mn-Tox O5 | 88.1 |
| H60N*^ε^*^2^-Mn- Wat20 | 171.3 | E138O*^ε^*^1^-Mn-E113O*^ε^*^1^ | 121.5 |
| H60N*^ε^*^2^-Mn-Tox O5 | 82.6 | E138O*^ε^*^1^-Mn-Wat20 | 84.3 |
| E113O*^ε^*^1^-Mn-Wat20 | 95.6 | E138O*^ε^*^1^-Mn-Tox O5 | 150.2 |
| E113O*^ε^*^1^-Mn-H60N*^ε^*^2^ | 87.6 | Tox O1-Mn-Wat20 | 89.5 |

Wat20 in the complex is equivalent to Wat13 in a substrate-free TxDE.
