# Supplementary figures and images for: Structural and Functional Analysis of Phytotoxin Toxoflavin-Degrading Enzyme
Source: PLoS One. 2011 Jul 25;6(7):e22443. doi: 10.1371/journal.pone.0022443 (PMC3143149; doi:10.1371/journal.pone.0022443)

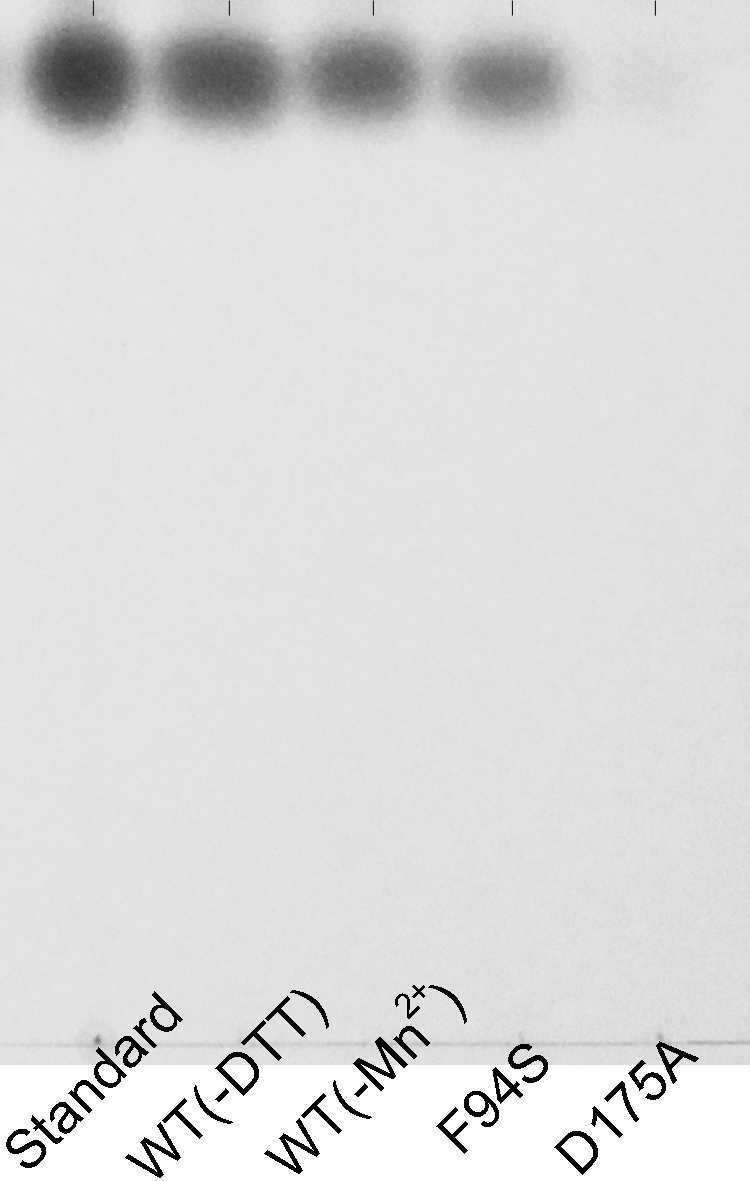

Supplement: Figure S1 — Thin-layer chromatographic analysis of toxoflavin degradation under various conditions. The enzyme reaction was carried out using three different enzymes: wild-type enzyme (WT), TxDE with the F94S mutation, and TxDE with the mutation D175A. For the reaction in the absence of DTT or Mn2+, the purified WT enzyme was dialyzed against buffer in the presence of 10 mM EDTA, and then DTT or Mn2+ was added. The “Standard” lane is toxoflavin in the absence of any other components. Toxoflavin was degraded by D175A mutant enzymes, but not by the F94S mutant enzyme, as well as in the absence of DTT or Mn2+. All reactions were carried out under aerobic conditions. (TIF) [file pone.0022443.s003.tif]

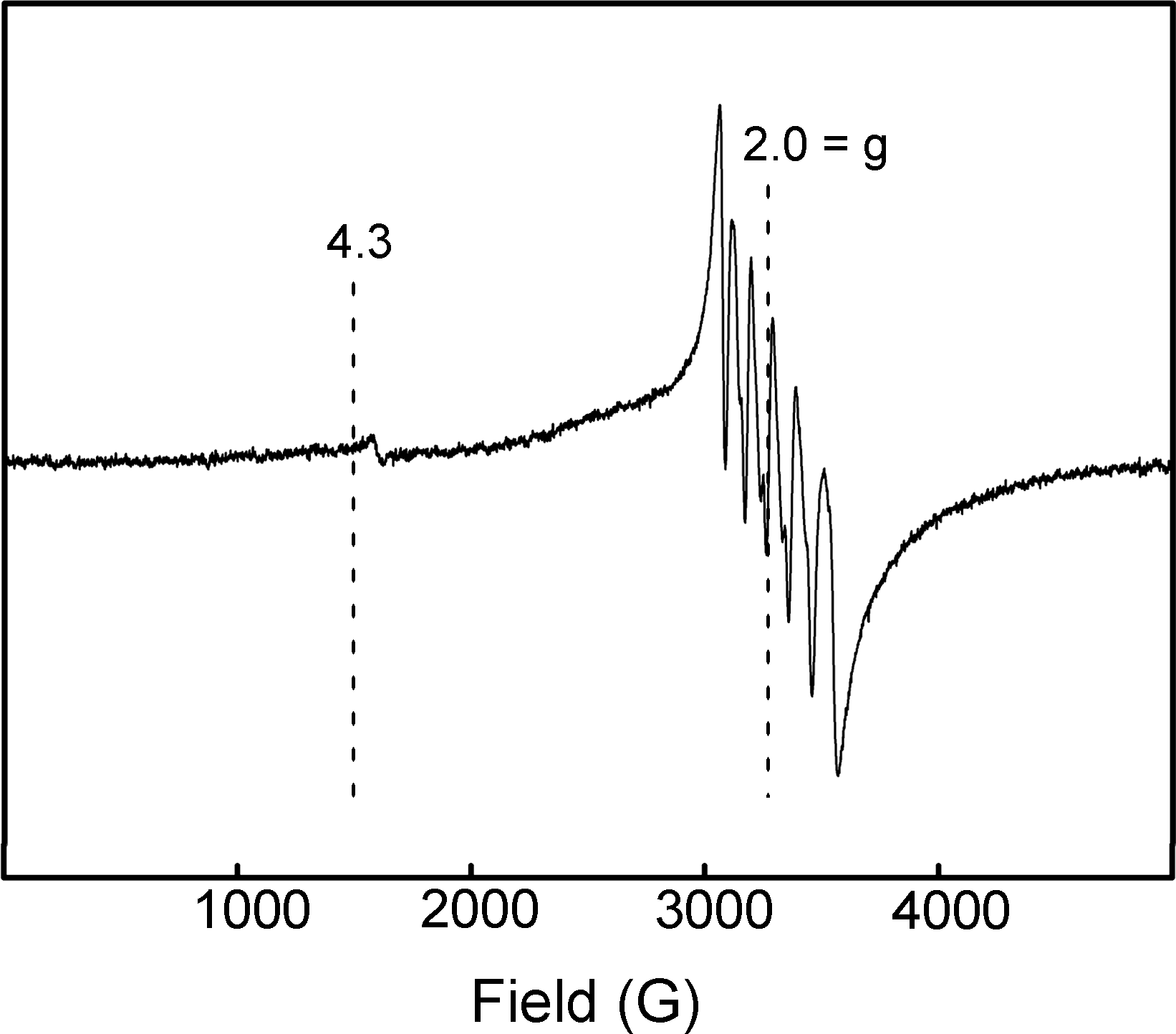

Supplement: Figure S2 — EPR spectrum of the purified TxDE. Sample contains 290 uM TxDE. EPR parameters: 100 K, 1-mW microwave power at 9.18 GHz, modulation amplitude 3.2G. (TIF) [file pone.0022443.s004.tif]

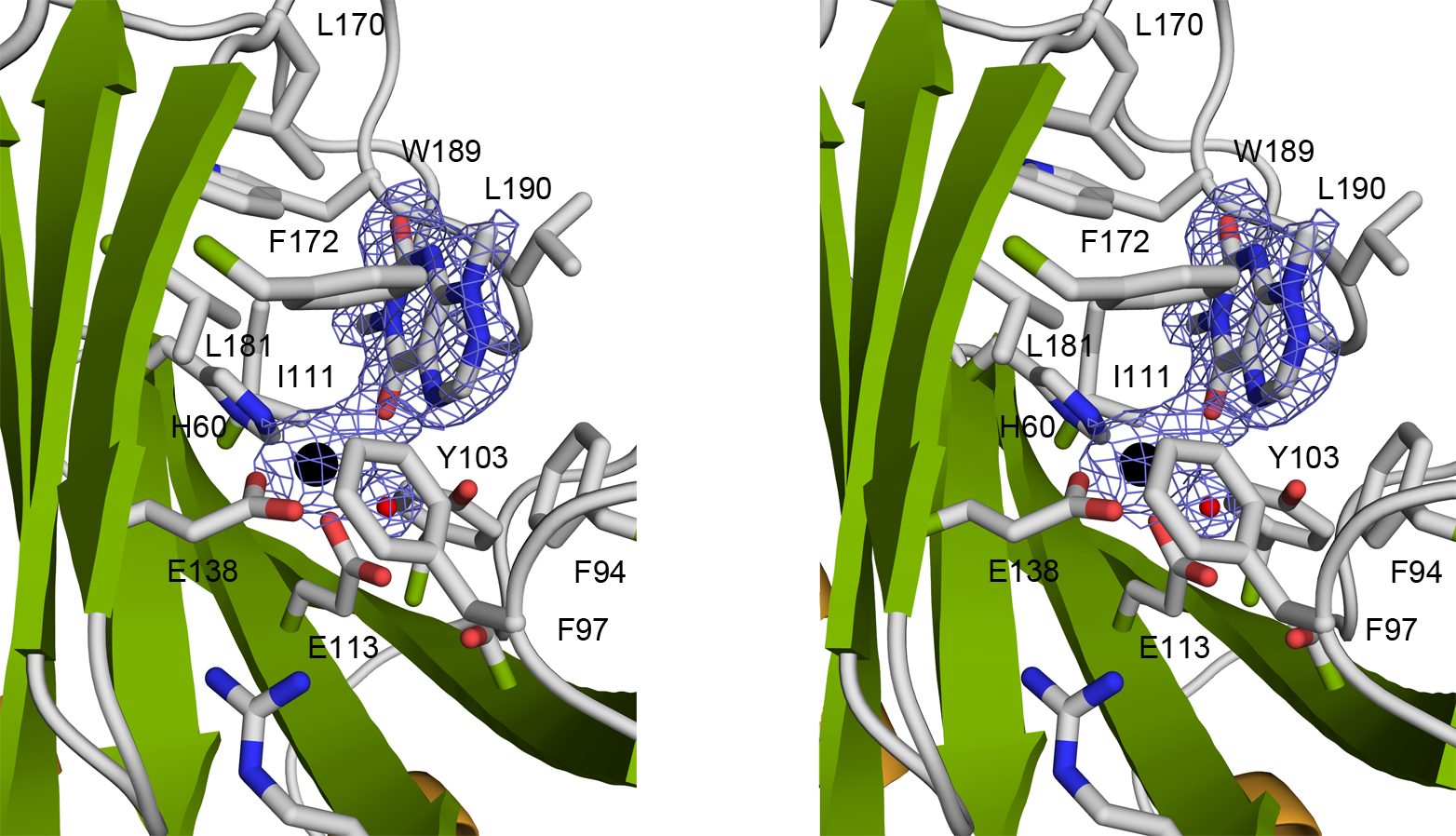

Supplement: Figure S3 — Stereoscopic view of the active site of the TxDE–toxoflavin complex. This view, obtained by a rotation of about 90° along the vertical axis of Figure 4A , illustrates that the possible sixth coordinating ligand is missing in this complex. The electron density of 2Fo-Fc contoured at 1 σ is shown for a bound Mn(II) (black sphere), water molecule (red sphere), and toxoflavin molecule. (TIF) [file pone.0022443.s005.tif]

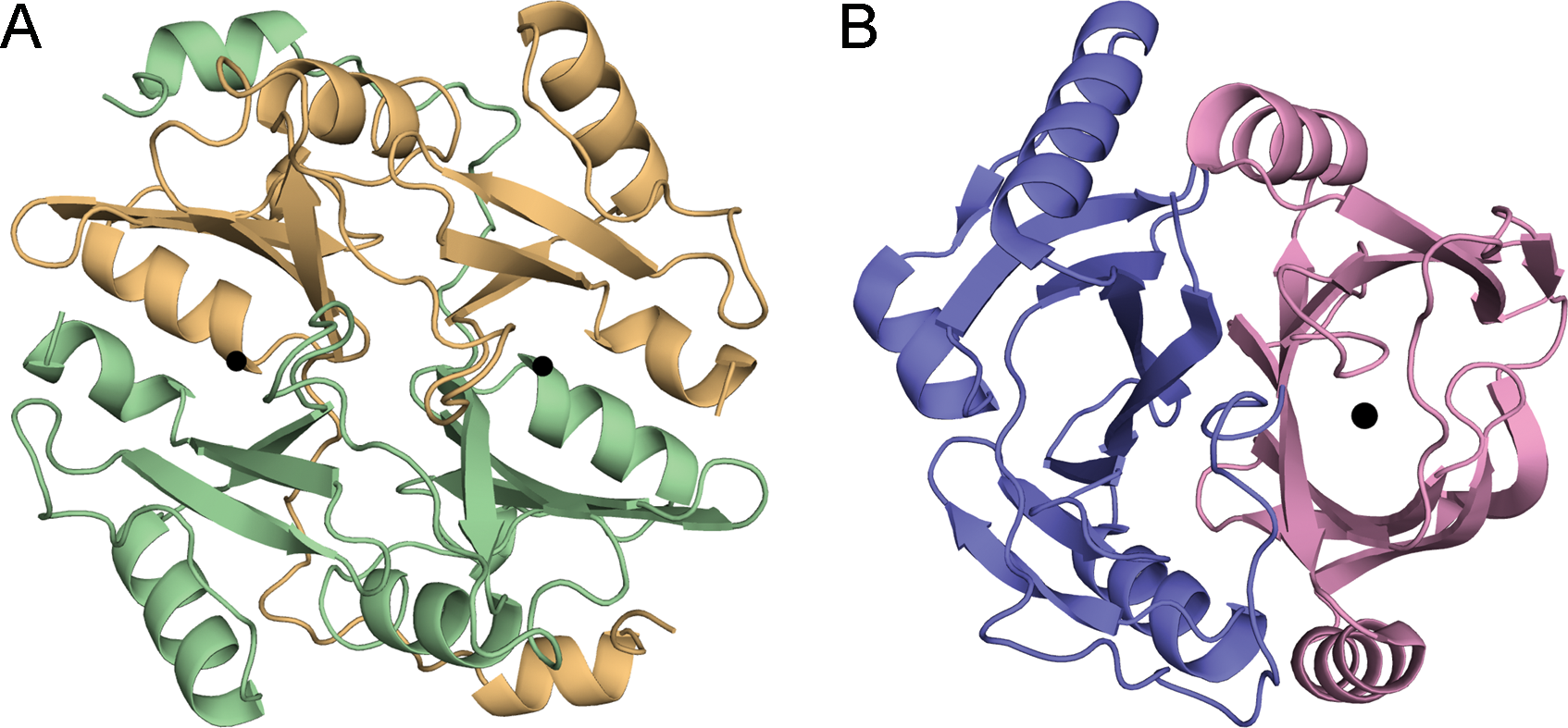

Supplement: Figure S4 — Overall structure of glyoxalase and 2,3-dihyroxybiphenyl 1,2-dioxygenase (DHBD). (A) As described in the text, a dimer of glyoxalase (PDB ID 1FRO) [37] generates two independent active sites at the intersubunit interface. Each monomer is indicated in a different color, and the active sites are presented with a bound metal ion (black sphere). (B) The structure of monomeric DHBD (PDB ID 1HAN) [23] was similar to that of TxDE in this study. Each domain is colored differently. In each domain, two sequentially ordered βαβββ motifs form continuous β-stands by edge-to-edge interactions. The C-terminal active site is shown with a bound metal ion (black sphere). (TIF) [file pone.0022443.s006.tif]

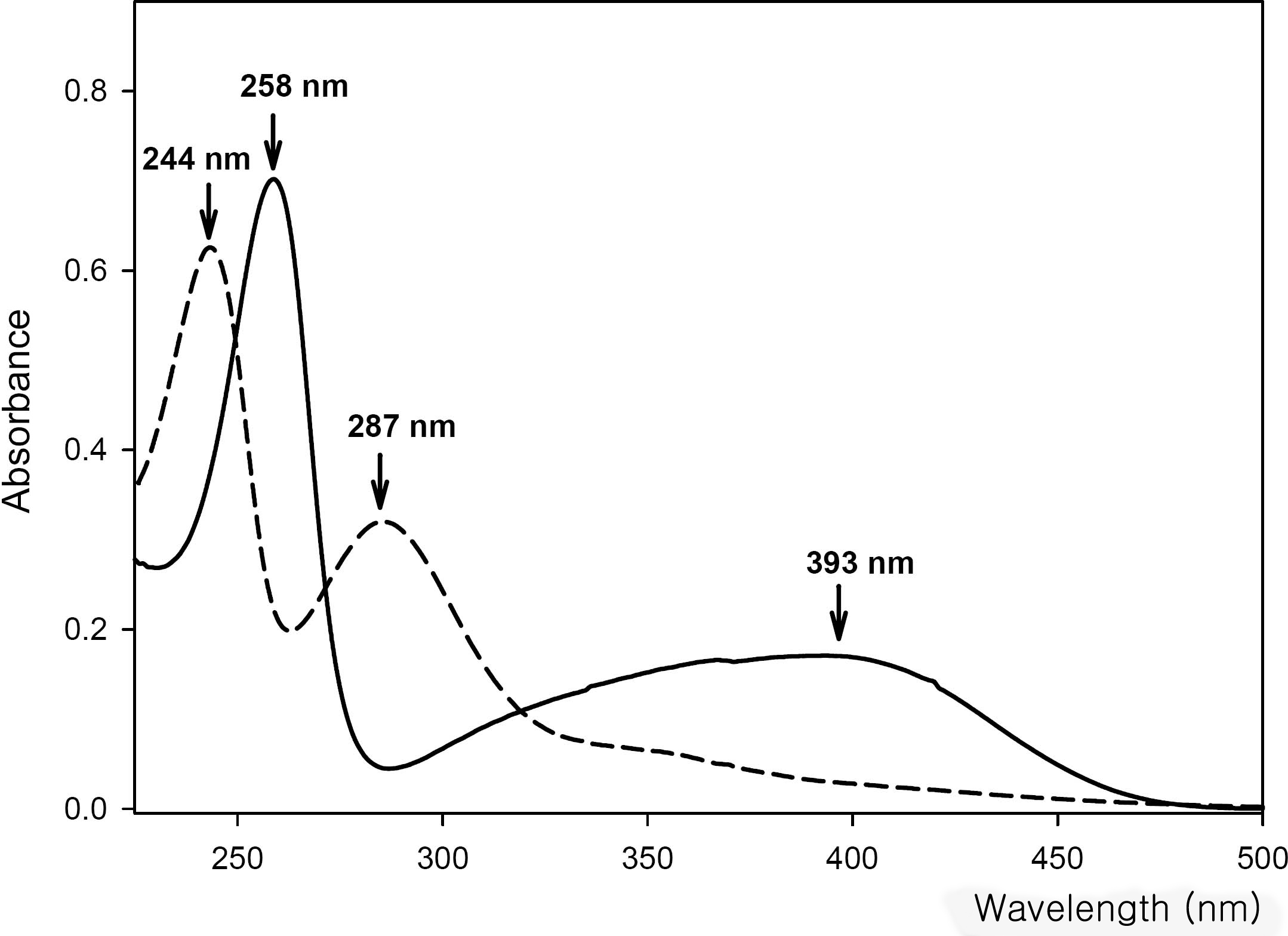

Supplement: Figure S5 — UV-Vis absorption spectra of toxoflavin in the absence and presence of DTT. Two different absorption spectra of toxoflavin (25 µM), which was dissolved in 50 mM HEPES, pH 6.8, and 10 µM MnCl2, were recorded under aerobic conditions. In the absence of DTT (solid line), toxoflavin exhibits two absorption peaks, at 258 and 393 nm. Upon the addition of 2 mM DTT (dashed line), two peaks appeared, at 244 and 287 nm. The absorption peak at 287 nm corresponds to that of the oxidized form of DTT (i.e., 1,2-dithiane-4,5-diol; DTD), and its absorbance varies according to the concentration of DTT used in the experiment. The peak at 244 nm was later identified by NMR spectroscopy as that of reduced toxoflavin (i.e., 4,8-dihydrotoxoflavin) (Figure S6); it remained stable only in the presence of DTT. After the DTT was exhausted, the spectrum of 4,8-dihydrotoxoflavin changed into that of toxoflavin (solid line) owing to oxidation by adventitious air or bubbled oxygen, with an additional absorbance shoulder at 287 nm for DTD. At this stage, toxoflavin was no longer degraded by the TflA enzyme, unless additional DTT was added to the reaction mixture, strongly suggesting that the reduced form of toxoflavin is the true substrate for the enzyme. (TIF) [file pone.0022443.s007.tif]

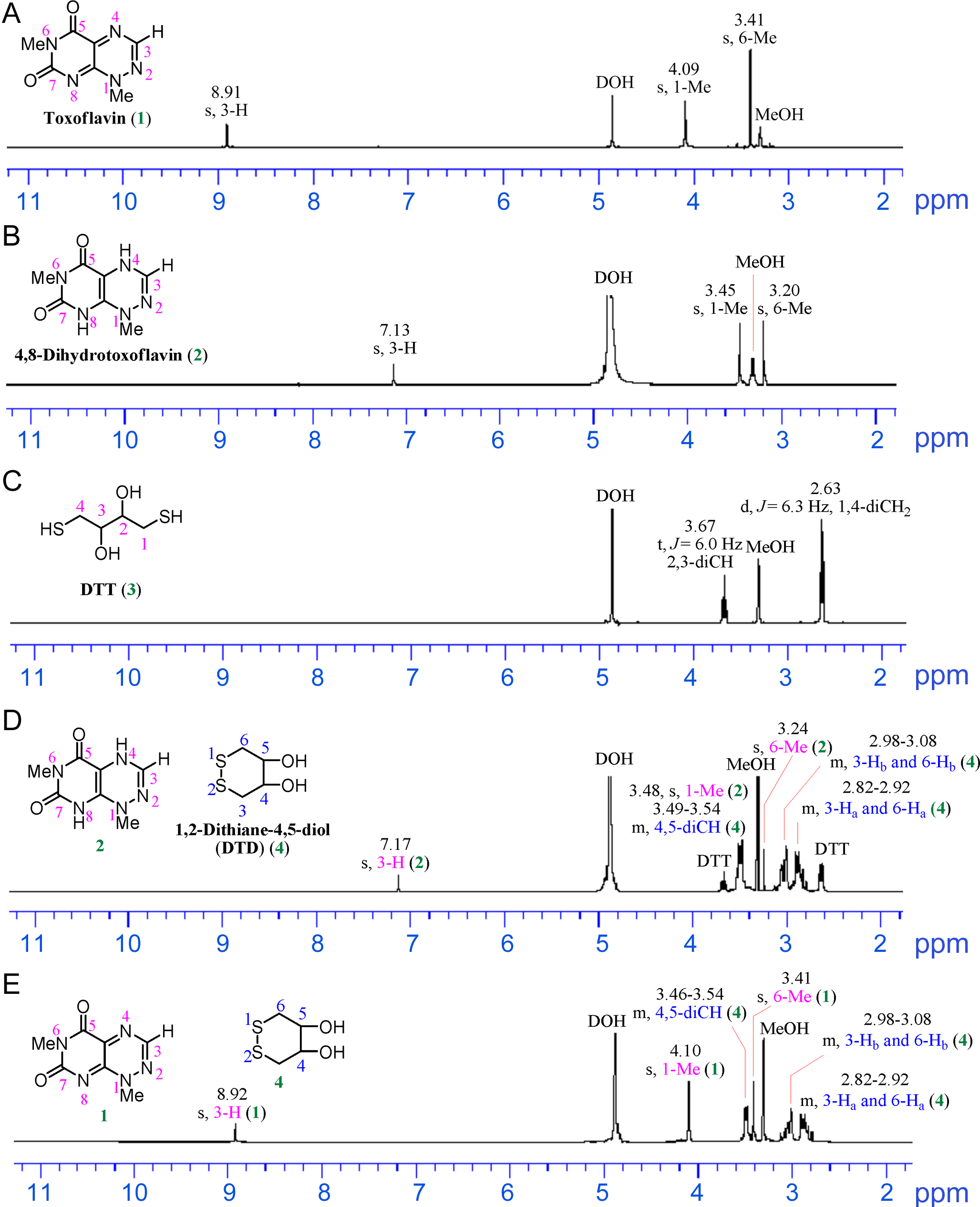

Supplement: Figure S6 — 1H-NMR experiments in deuterated methanol (99% CD3OD). 1H-NMR chemical shifts under aerobic conditions at 22°C for (A) pure toxoflavin (1), (B) pure 4,8-dihydrotoxoflavin (2), and (C) pure DTT (3) are shown with peak assignments for each proton in the compounds. (D) The 1H-NMR experiment was performed in deuterated methanol after a 10-min reaction of toxoflavin (1) with an equimolar amount of DTT (3) at 22°C under aerobic conditions. A chemical shift analysis indicated that the reaction mixture contained predominantly 4,8-dihydrotoxoflavin (2) and DTD (4) with a small quantity of DTT (3), consistent with the results of the UV-Vis spectroscopic analysis shown in Figure S5. (E) After the reaction of toxoflavin (1) with an equimolar amount of DTT (3) in deuterated methanol at 22°C for 10 min under aerobic conditions, oxygen was bubbled into the reaction mixture for 1 min. The analysis indicated that all DTT (3) was converted into DTD (4) and 4,8-dihydrotoxoflavin (2) was converted into toxoflavin (1) by oxidation, again consistent with the results of the UV-Vis spectroscopic analysis shown in Figure S5. (TIF) [file pone.0022443.s008.tif]
